# Efavirenz restored NMDA receptor dysfunction and inhibited epileptic seizures in *GluN2A/Grin2a* mutant mice

**DOI:** 10.3389/fnins.2023.1086462

**Published:** 2023-03-02

**Authors:** Teng Zhao, Rui Zhong, Xinyue Zhang, Guangjian Li, Chunkui Zhou, Shaokuan Fang, Ying Ding, Weihong Lin

**Affiliations:** ^1^Department of Neurology, The First Hospital of Jilin University, Changchun, China; ^2^Department of Radiology, The First Hospital of Jilin University, Changchun, China

**Keywords:** efavirenz, NMDA receptor, epilepsy, loss of function, GluN2A/GRIN2A-V685G mutation, seizure

## Abstract

**Introduction:**

N-methyl-D-aspartate receptor (NMDAR) is one of the main receptor of the excitatory neurotransmitter glutamate in the brain, which is the key determinant of the excitatory/inhibitory balance of neural network. GluN2A/GRIN2A is one of the subunits of NMDAR and plays an important role in epilepsy. Approximately 78% of patients with *GluN2A/Grin2a* mutations have epilepsy, and the underlying mechanism of this association is not well characterized.

**Methods:**

We constructed a mouse model of hyperthermic seizure, and conducted *in vitro* and *in vivo* electrophysiological and behavioral studies to clarify the pathogenic characteristics and mechanism of GluN2A/GRIN2A-V685G mutation. In addition, the drug efavirenz (EFV), which is used to treat HIV infection, was administrated to mutant animals to assess whether it can restore the loss of function.

**Results:**

Mutant mice showed no significant change in the mRNA or protein expressions of NMDAR compared with wild type (WT) mice. Mice with GluN2A/GRIN2A-V685G mutation exhibited shorter latency to seizure, increased frequency of seizure-like events, decreased peak current and current area of NMDAR excitatory postsynaptic current, and decreased event frequency of micro-inhibitory postsynaptic current, compared to WT mice. They also exhibited decreased threshold, increased amplitude, increased input resistance, and increased root number of action potential. EFV administration reversed these changes. The loss-of-function (LoF) mutation of NMDAR changed the excitatory/inhibitory balance of neural network, rendering animal more prone to seizures.

**Discussion:**

EFV was indicated to hold its potential in the treatment of inherited epilepsy.

## 1. Introduction

Epilepsy is one of the most common neurological diseases in the world that can affect people of all age-groups. Currently, an estimated 50 million patients are affected by epilepsy worldwide (Herbst et al., 2018). Epilepsy is characterized by recurrent episodes of seizures, which result from abnormal neuronal networks and excessive electrical discharge in brain cells (O’Dell et al., 2012). The type and form of seizures varies depending on the location and speed of the spread of abnormal discharge in the brain. These may affect body movement (partial or whole-body), sensation (visual, auditory and/or gustatory), mood, and cognition (Bromfield et al., 2006). Approximately half of all patients with epilepsy show no known underlying cause of this disease. Brain damage, congenital abnormalities, head injury, stroke, brain infection, and tumor can all cause epilepsy (Herbst et al., 2018). Although the exact mechanism of epileptic seizures is unknown, some cellular and network changes have been discovered (Cavanna and Monaco, 2009; Janigro and Walker, 2014). Alterations of ion homeostasis, energy metabolism, receptor function, and transmitter uptake have all been implicated in the pathophysiology of epilepsy (Cloix and Hévor, 2009; Crépel and Mulle, 2015; Raimondo et al., 2015; Bazzigaluppi et al., 2017; Falcón-Moya et al., 2018; Engel et al., 2021; Negrete-Díaz et al., 2022).

Imbalance between excitatory and inhibitory synaptic signals is an important cause of epilepsy. N-methyl-D-aspartate receptor (NMDAR) is the main receptor of the excitatory neurotransmitter glutamate in the brain (Li and Tsien, 2009). As a ligand-gated ion channel, it mediates the production and transmission of excitatory postsynaptic current (EPSC) inside the synapse, and plays a role in the presynaptic release of γ-aminobutyric acid (GABA) from GABAergic interneurons outside the synapse, thus affecting the production of inhibitory postsynaptic current (IPSC) (Gonzalez-Burgos and Lewis, 2008; Sihra and Rodríguez-Moreno, 2011). Therefore, NMDAR is a key determinant of the excitatory/inhibitory balance of neural networks (Rodríguez-Moreno and Sihra, 2011; Sihra and Rodríguez-Moreno, 2013; Falcón-Moya et al., 2019; Falcón-Moya and Rodríguez-Moreno, 2021). As a heterotetramer, NMDAR is comprised of two GluN1 subunits and two GluN2 subunits (Franchini et al., 2020). GluN1 subunit, for glycine-binding, is encoded by the *GRIN1* gene and is ubiquitously expressed in the brain. GluN2 subunits, for glutamate-binding, have four subtypes 2A, 2B, 2C, and 2D, encoded by the *GRIN2A*, *2B*, *2C*, and *2D* genes, respectively. These subunits show different expression patterns. GluN2A and GluN2C subunits are predominantly expressed in the postnatal period and in adults, whereas GluN2B and GluN2D subunits are mainly expressed in the prenatal period (Lakhan et al., 2013; Sanz-Clemente et al., 2013). Binding of both glutamate and glycine is required for the activation of NMDAR channels, which results in the opening of the channels, followed by the elevation of intracellular calcium levels and membrane depolarization (Lipton, 2004; Andrade-Talavera et al., 2013; Geoffroy et al., 2021). Some clinical studies and cell-level receptor function analysis have implicated gene mutation in NMDAR, either gain-of-function (GoF) or loss-of-function (LoF) mutation, in the causation of epilepsy (Olivares et al., 2012). GoF mutation of NMDAR can cause increase in EPSC, leading to an imbalance in the excitatory/inhibitory discharges in the neuronal network. LoF mutations are believed to weaken IPSC by inhibiting the release of GABA from the presynaptic membrane of GABAergic neurons (XiangWei et al., 2018), or by postsynaptic mechanism (Gu et al., 2016), leading to epilepsy. Other studies have shown that some mutations may lead to a decrease in NMDAR expression levels in cell surface, thereby reducing function (Gao et al., 2017). However, the pathogenetic mechanism by which GoF or LoF mutation of NMDAR gene causes epilepsy is not well characterized. Thus, many experimental and clinical researches have focused on the mutations and functions of NMDAR to unravel its role in epilepsy.

Recently, some drugs used for the treatment of other diseases, especially Alzheimer’s disease, have shown good results when applied as anti-epileptic treatment. For example, use of memantine in the treatment of epilepsy caused by NMDAR GoF mutations showed good outcomes (Swanger et al., 2016). Some allosteric modulators were shown to reverse the abnormal NMDAR function caused by some gene variations (Xu and Luo, 2018). Screening the effective positive allosteric modulators (PAMs) in NMDAR mutations at cell and animal levels can provide important theoretical and practical support for clinical precision treatment. Efavirenz (EFV), which is an anti-HIV drug, has recently been used in the research on Alzheimer’s disease (Pikuleva et al., 2018). EFV can increase the activity of CYP46A1, thereby increasing the level of 24(S)-hydroxycholesterol (24(S)-HC) in the brain and serum (Mast et al., 2017). Studies have shown that 24(S)-HC can restore the function of NMDAR caused by GluN2A/GRIN2A-V685G LoF mutation (Tang et al., 2020). Whether EFV can be used in the treatment of epilepsy and the potential related mechanisms are not clear.

In the present study, we selected GRIN2A-V685G, a relatively common mutation site confirmed at the cellular level and in clinic settings, as the research target, and conducted electrophysiological and behavioral studies in mutant animals *in vivo* and *in vitro*. The objective was to clarify the pathogenic characteristics and mechanism of this gene mutation in animals. In addition, we investigated the effect of EFV on the function of NMDAR and epileptic seizure in mutant animals. Our findings may provide a theoretical basis for further research in the realm of precision therapy for hereditary epilepsy and other related genetic diseases.

## 2. Materials and methods

### 2.1. Animals

All experimental procedures involving mice were approved by the Animal Welfare and Research Ethics Committee of The First Hospital of Jilin University (Permit Number: 20200721). GluN2A/GRIN2A-V685G mutant mice were generated by using C57BL/6N mice. *GluN2A/Grin2a* mutation was done at the 685th amino acid from Valine (Val, V) to glycine (Gly, G). The primers used were 5′CGATTTGGGACAGGGCCTAATGGAAG 3′ and 5′ CTTCCATTAGGCCCTGTCCCAAATCG3′. The mutant coding sequence (CDS) was inserted into the pCAG vector. After isolation and purification, the vector bearing mutant *GluN2A/Grin2a* (∼7.6 kb) was injected into C57BL/6N mouse embryos, and the embryos were transplanted into the oviduct of surrogate recipient mice. Genotype identification was completed approximately 2 weeks after birth. The primers used for genotype identification by tail-cutting method were 5′-CGCCTCTGTCTGGGTGATGATGTTT GT-3′ and 5′-CTTCCATTAGGCCCTGTCCCAAATCG-3′ in PCR, and the PCR product was 405 bp.

According to the principle of 50/50 male and female, the GluN2A/GRIN2A-V685G mutant mice were randomly divided into mutation (LoF) group and mutation treatment (LoF + EFV) group. Mice of the same generation without GluN2A/GRIN2A-V685G mutation were randomly divided into wild-type (WT) group and wild-type treatment (WT + EFV) group. There were 14 mice in WT group, 12 in LoF group, 12 in WT + EFV group, and 12 in LoF + EFV group. EFV was administered by dissolving in drinking water at the age of 4 weeks (0.42 g/L) (Mast et al., 2017) for 14 days.

### 2.2. Behavioral evaluation

All mice (age: 8 weeks) were monitored for 1 week for any spontaneous epileptic seizure by videography. In case of spontaneous epileptic seizures, the number, duration, and the intensity of seizures were recorded. If there was no spontaneous epileptic seizure, hyperthermic seizure model was established in each group under the same conditions (Ullal et al., 1996). Mice were placed in hot water bath at 44.5–45°C for 4 min or until epileptic seizure. The latency to seizure (usually myoclonus), seizure duration (from onset to the first return to normal and emergence of consciousness), and seizure intensity were recorded. Seizure intensity was categorized into five levels: level 0, no convulsive behavior; level 1, facial clonus; level 2, nodding; level 3, anterior clonus; level 4, rearing behavior (standing, extending hind legs, enhanced tone of voice); level 5, standing up or down. The scoring of seizure was performed by an investigator who was blinded to this study.

### 2.3. *In vivo* EEG detection

After behavioral evaluation, 6 mice (3 male and 3 female) were randomly selected from each group and subjected to *in vivo* cortical EEG. The mice were anesthetized by intraperitoneal injection of 10% chloral hydrate (0.4 ml/kg). The cortex of mice was stereotactically inserted with EEG recording electrode (2.5–3.5 mm, 1.2 mm). Two silver-wire electrodes were inserted into the muscles at the back of the neck to record the electrical activity. All animals were allowed to rest for 5–7 days after surgery.

Cortical EEG signals were collected using pinnacle preamplification system (USA) and PowerLab recording system (Australia). The signal filtration was band-pass 0.1–30 Hz with a sampling rate of 400 Hz. Cortical EEG was recorded in the awake state for 1–2 h a day for 7 consecutive days. The number of seizure-like events (SLEs) was also recorded for 7 days.

### 2.4. Patch-clamp recordings in brain slices

After anesthetization, the mouse brain was quickly removed and placed in artificial cerebrospinal fluid (ACSF, containing 234 mM Sucrose, 2.5 mM KCl, 1.25 mM NaH_2_PO_4_.2H_2_O, 25 mM NaHCO_3_, 25 mM D-glucose, 0.5 mM CaCl_2_.2H_2_O, and 10 mM MgSO_4_) (0–4°C) supplied with 95% O_2_ and 5% CO_2_. The hippocampal tissue was transected by vibrating section (Leica, VT 1000 S, Germany) with a thickness of 300 μm. During the sectioning of the hippocampus, the gas was continuously infused in the petri dish and the specimen tank. The brain slices were then incubated in ACSF at 32°C for 30 min, followed by incubation for 30 min at 22–23°C. Subsequently, the brain slices were transferred one by one to the recording tank with ACSF flow (31 ± 1°C, 6 ml/min).

The intracellular recording solution includes 135 mM Cs-meth, 10 mM NaCl, 2 mM MgCl_2_.6H_2_O, 10 mM HEPES, 10 mM EGTA, 2 mM Na_2_-ATP, and 0.2 mM Na_2_-GTP. The capillary glass tube was drawn into stimulus electrode and recording electrode by microelectrode drawing instrument, and the stimulation electrode was placed approximately 200 μm away from the cell. After immersion of the recording electrode into the liquid, positive pressure was applied, and the tissue was blown away while the recording electrode was injected downward until the electrode contacted the cell membrane. Negative pressure was applied for suction to form GΩ sealing. After GΩ sealing was formed, rapid capacitance compensation was performed, and then negative pressure was continued to absorb the membrane. After the membrane was broken under negative pressure, a voltage stimulation of 5 mV was recorded in gap-free mode after 3–5 min of cell stability.

The stimulation and NMDA current recording in the hippocampal CA1 region were as follows. A total of 20 μM bicuculline, 20 μM DNQX were added to the external fluid. The membrane potential was clamped at +50 mV, and the appropriate stimulation intensity was provided until the NMDA current reached about 100 pA. The physiological signals were then recorded for 5 min or until finding the maximum value on 3–5 roots with stability. The interval of each trace was 30 s. The change of series resistance before and after recording was also recorded.

Microinhibitory postsynaptic current (mIPSC) in GABAergic interneurons in the hippocampal CA1 region was recorded as follows. A total of 1 μM TTX, 20 μM DNQX, and 20 μM DAP-5 were added into the extracellular fluid. Clamping voltage was −70 mV. The recording electrode was close to the cell and negative pressure was applied to the cell to form high resistance sealing (>1 GΩ). After sealing, rapid capacitor compensation was performed. Gap-free mode was used for recording for 10 min after stabilization. The experimental data were analyzed offline with Clampfit10.6 to calculate the current amplitude and discharge frequency.

The stimulation and the action potential recording of pyramidal neurons in the hippocampal CA1 region was as follows. The ACSF contains 125 mM NaCl, 2.5 mM KCl, 1.25 mM NaH_2_PO_4_.2H_2_O, 25 mM NaHCO_3_, 10 mM D-Glucose, 2 mM CaCl_2_.2H_2_O, 1.5 mM MgSO_4_. The electrode fluid contains 140 mM K-gluconate, 2 mM MgCl_2_, 10 mM HEPES, 8 mM KCl, 2 mM Na_2_-ATP, and 0.2 mM Na_2_-GTP, pH 7.3. After the membrane was broken, the negative voltage was removed and the membrane capacitance was compensated using the “Whole Cell” automatic compensation function. By using the IC-clamp mode, the action potential was induced from −50 to 200 Pa in incremental steps of 10 Pa.

The following criteria are used to judge if the data is acceptable: electrode resistance <5 MΩ; sealing resistance >1 GΩ; series resistance <25 GΩ.

### 2.5. Real-time PCR

Total RNA was extracted from mouse hippocampus using TRIzol Reagent (Sigma-Aldrich, USA) and reverse-transcribed into cDNA using HiScript III 1st Strand cDNA Synthesis Kit (Vazyme, Nanjing, China). Primers are listed in Table 1. Real-time PCR was performed in a total reaction volume of 20 μl, containing 2 μl cDNA, 1 μL forward primer, 1 μl reverse primer, 0.4 μl ROX Reference DyeII, 10 μl 2 × SYBR Premix Ex Taq II, and 1 μl nuclease-free water. The amplification program was as follows: 30 s at 95°C; 40 cycles of 5 s at 95°C, 30 s at 60°C, 30 s at 72°C, and the dissolution curve. The relative value RQ (RQ = 2^–ΔΔ*Ct*^/mean value of WT group) was calculated using ΔΔCt method. GAPDH was used as internal reference.

**TABLE 1:** Primers for real-time PCR.

Primer name	Sequence (5′-3′)
mNR2A-F	ACCATTGGGAGCGGGTACAT
mNR2A-R	CCTGCCATGTTGTCGATGTC
mGAPDH-F	AATGTGTCCGTCGTGGATCTGA
mGAPDH-R	GATGCCTGCTTCACCACCTTCT

### 2.6. Western blot

Protein samples were extracted from mouse hippocampi with RIPA lysis (Beyotime, China) and the concentration was determined by BCA assay (Beyotime, China). After separation on 12% SDS-PAGE gels, the proteins were transferred on to PVDF membranes (Millipore, USA). The membranes were then blocked in 5% non-fat milk, incubated with antibody (1:2,000 dilution, Abcam, UK), and washed sequentially. After incubation with horseradish peroxidase-linked secondary antibody (1:4,000 dilution, ZSGB-BIO, China), the membranes were washed, reacted with enhanced chemiluminescence detection reagents (Applygen, China), and visualized after exposure to Kodak X-ray film. Image J was used for quantitative analysis, and the relative expression levels were calculated.

### 2.7. Data analysis

Data analyses were performed using Clampfit 10.6 and GraphPad 8. All experimental results are presented as mean ± standard error. Differences among groups were analyzed using one-way analysis of variance (ANOVA) followed by Tukey’s *post-hoc* test. *P* values < 0.05 were considered indicative of statistical significance.

## 3. Results

### 3.1. No change in the expression of NR2A in GluN2A/GRIN2A-V685G mutant mice

**FIGURE 1:**
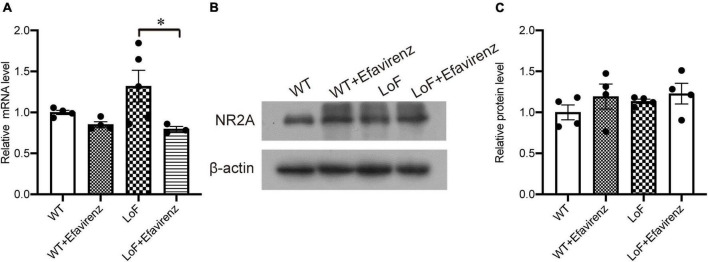
mRNA and protein expressions of NR2A in the hippocampi of GRIN2A-V685G mutant mice. GRIN2A-V685G mutant mice were established on C57BL/6N background. The mRNA and protein levels of NR2A were assessed at the age of 8 weeks. **(A)** mRNA level, **(B)** protein level, **(C)** relative protein level. *P < 0.05.

In constructing mutant mice, the success rate of embryo transfer was 16–33%. Compared with control mice, there were no significant differences in the appearance, weight, food intake, or behavioral performance in cognitive and depression tests; however, some differences were observed in the test for anxiety (data not shown). After establishment of GluN2A/GRIN2A-V685G mice, we firstly detected the expression of NR2A in mutant mouse brain. Results of real-time PCR showed no significant difference in the mRNA expressions of NR2A in the hippocampal CA1 region among the mice of all groups (Figure 1A), except the level of NR2A mRNA in the mice of LoF group was higher than that of LoF + EFV group significantly. The protein levels of NR2A in the mouse hippocampal CA1 region of each group were also not changed (Figures 1B, C). GluN2A/GRIN2A-V685G mutation and EFV showed no influence on the protein expressions of NR2A.

### 3.2. Establishment of epileptic model in mutant mice

**FIGURE 2:**
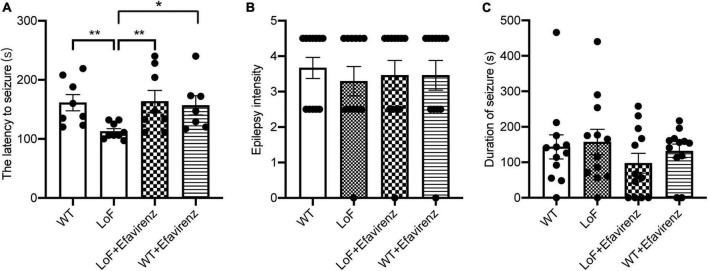
The latency, intensity, and duration of seizure in mice induced hyperthermically. Mice were subjected to hyperthermic seizure test. The latency to seizure **(A)**, seizure intensity **(B)**, and seizure duration (from onset to the first return to normal and emergence of consciousness) **(C)** were recorded. *P < 0.05; **P < 0.01.

Spontaneous seizures were not observed in mice in any of the groups before hyperthermic intervention. After the establishment of hyperthermic seizure model, there were significant differences in the latency to seizure between mice in LoF group and WT group (*P* = 0.003), and between mice in LoF group and LoF + Efavirenz group (*P* = 0.006). GluN2A/GRIN2A-V685G mutation was more likely to induce hyperthermic convulsions than WT, while EFV treatment was found to delay the seizure occurrence in mutant mice (Figure 2A). No significant differences in the intensity and duration of convulsions were observed among the mice of different groups (Figures 2B, C). However, the duration of seizures in the LoF group was longer than that in the WT group, while the duration of seizures in the LoF + EFV group was shorter than that in the LoF group.

### 3.3. Change in *in vivo* EEG

**FIGURE 3:**
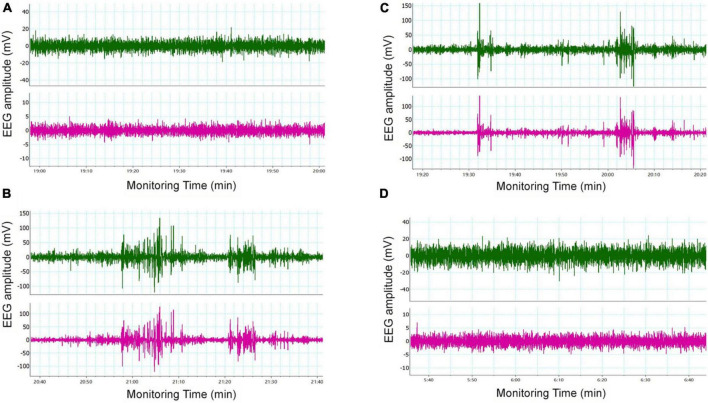
Typical cortical EEG in mice. Mice were subjected to *in vivo* EEG detection and cortical EEG signals were collected. Green is frontal lobe electrode, red is parietal lobe electrode, and blue is neck EMG. **(A)** WT group, **(B)** LoF group, **(C)** LoF + EFV group, and **(D)** WT + EFVgroup.

Seizure-like events (SLEs) were detected by cortical EEG. SLEs are spontaneous, paroxysmal, high-amplitude spikes, the maximum amplitude of which can reach 100 mV. Varying number of low amplitude (20–60 mV) spikes can occur during SLEs. In our study, 5 out of 6 mice in the LoF group showed spontaneous SLEs. Mice in the LoF group exhibited 23 SLEs in total, each lasting for 3–10 min. Among these, a mouse showed a single SLE lasting 10 min, manifesting as nodding (level 2 epileptic seizure). Besides, the mice in this group showed a small amount of spontaneous low amplitude spikes (6–12 within 1 h) during the interictal period (Figure 3). In the LoF + EFV group, 3 of 6 mice showed spontaneous SLEs, 7 times in total. The amplitude of SLEs was only two times higher than the baseline, and lasted only 2–5 min, with occasional small spikes (1–5 within 1 h) in the interphase. No SLEs were detected in WT group or WT + EFV group within 7 d of monitoring, and small spikes were occasionally observed in the interphase (1–5 within 1 h).

### 3.4. Change in features of pyramidal neurons

**FIGURE 4:**
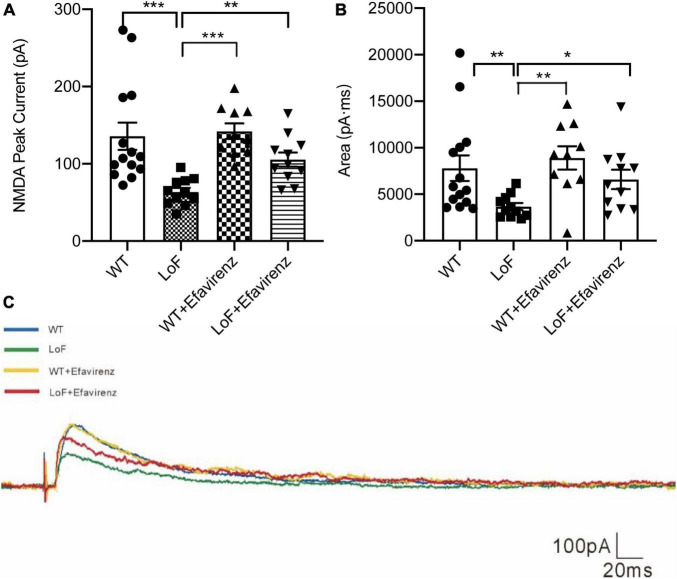
The peak current and current area of NMDAR in pyramidal cells in hippocampal CA1 region of mice. The mouse brain slices were used for patch-clamping recording in pyramidal cells in the hippocampal CA1 region of mice. The peak current **(A)** and current area **(B)** of NMDA current were recorded. The typical current are shown **(C)**. *P < 0.05; **P < 0.01; ***P < 0.001.

On comparing the peak current (amplitude) of NMDAR in pyramidal cells in hippocampal CA1 region, significantly higher peak current was observed in WT group and LoF + EFV group, compared to LoF group (*P* < 0.0001 and *P* = 0.005, respectively). However, mice in WT group and WT + EFV group did not show any significant difference in NMDA peak current (*P* = 0.672), neither those in WT group and LoF + EFV group (*P* = 0.384) (Figure 4A).

On comparing the current area of NMDAR in pyramidal cells of hippocampal CA1 region, similar results were observed; more current area was observed in WT group and LoF + EFV group, compared to LoF group (*P* = 0.005 and *P* = 0.032, respectively). However, there was no significant difference between WT group and WT + EFV group in this respect (*P* = 0.555) (Figures 4B, C). These results indicated that mice with GluN2A/GRIN2A-V685G mutation influenced the peak current and current area of NMDAR-mediated current, while EFV treatment can recover the changes.

**FIGURE 5:**
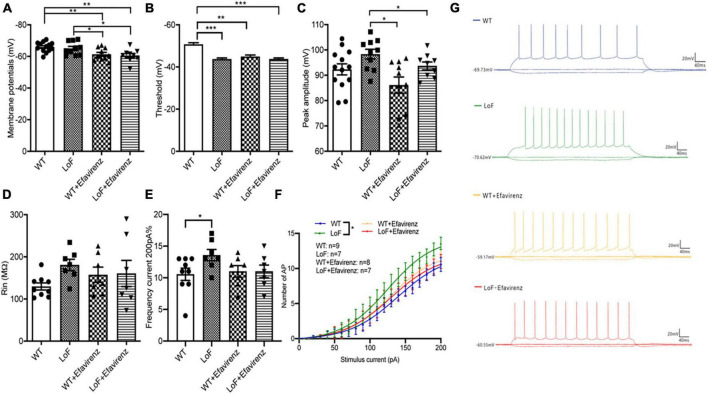
Comparison of action potential parameters of pyramidal neurons in hippocampal CA1 region of mice. Mouse brain slices were used for recording the action potential of pyramidal neurons in the hippocampal CA1 region. **(A)** Membrane potential, **(B)** threshold, **(C)** peak amplitude, **(D)** Rin, **(E)** the number of action potentials at 200 pA current generated by pyramidal neurons, **(F)** the number of action potential, and **(G)** representative recordings of AP in each group. *P < 0.05; **P < 0.01; ***P < 0.001.

Next, we compared the parameters of action potentials of hippocampal pyramidal neurons in CA1 region of mice. There were significant differences in action potential membrane potential of pyramidal cells in hippocampal CA1 region among mice of different groups (Figure 5A). The action potential threshold of pyramidal cells was significantly higher in mice of WT group than that of LoF groups (*P* < 0.0001). The action potential threshold was also significantly different between WT group and WT + EFV group (*P* = 0.001). But there was no significant difference in action potential threshold between LoF group and LoF + EFV group (*P* = 0.962) (Figure 5B). The peak amplitude of action potential of pyramidal neurons showed no significant difference between WT group and LoF group (*P* = 0.059). No significant difference in this respect was observed between WT group and WT + EFV group, but between LoF group and LoF + EFV group (*P* = 0.036) (Figure 5C).

On comparing the action potential input resistance of pyramidal neurons in hippocampal CA1 region of mice, no significant difference was observed in LoF group than in WT group (*P* = 0.268), while there was also no significant difference between WT group and WT + EFV group or between LoF group and LoF + EFV group (Figure 5D). These results indicated no significant increase in the input resistance of the mutant group during action potential.

On comparing the frequency and number of AP roots generated by hippocampal CA1 pyramid neurons in each group under the current of 200 pA, Higher frequency of AP was observed in LoF group than in WT group (*P* = 0.031), but no significant difference was shown between WT group and WT + EFV group or between LoF group and LoF + EFV group (Figures 5E–G). For the same current, the number of action potential generated in the mutant mice was more, while EFV tended to reduce the number, but the difference was not significant.

### 3.5. Change in features of GABAergic interneurons

**FIGURE 6:**
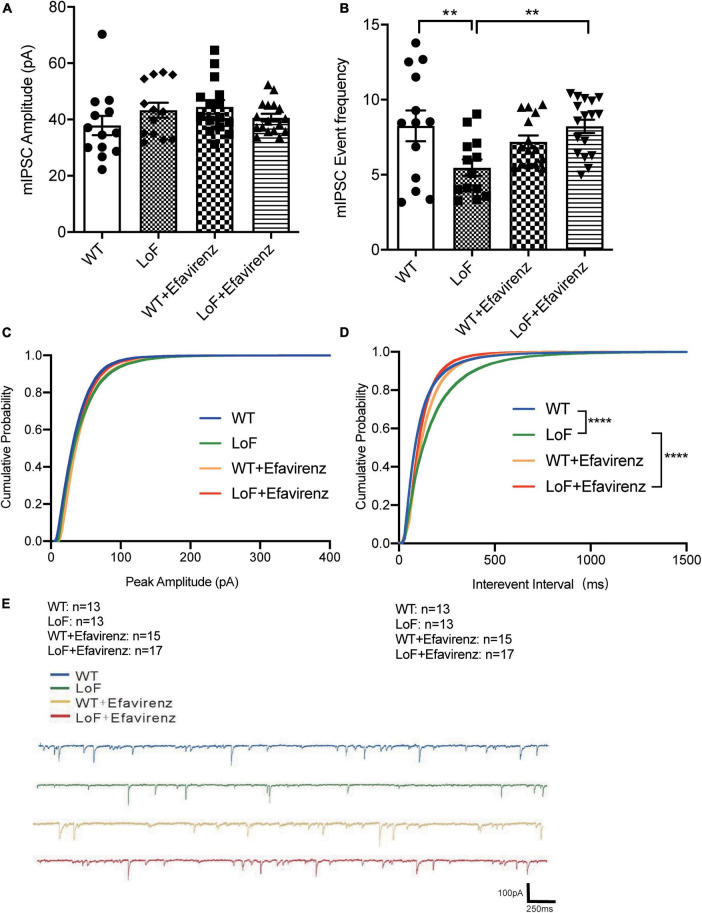
Comparisons of the amplitude and event frequency of mIPSC in hippocampal CA1 region of mice. Mouse brain slices were used for recording mIPSC in the hippocampal CA1 region. **(A)** Amplitude, **(B)** event frequency, **(C)** cumulative probability of peak amplitude, **(D)** cumulative probability of inter-event interval, and **(E)** the typical mIPSC in each group. **P < 0.01; ****P < 0.0001.

As contrary results were obtained from NMDA current of pyramidal cells and action potential of hippocampal pyramidal neurons in the CA1 region of mice, the peak amplitude of mIPSC in GABA interneurons in the hippocampal CA1 region of mice was measured (Figure 6). The results showed no significant difference among all the groups (Figure 6A).

On comparing the mIPSC event frequency of GABAnergic interneurons in hippocampal CA1 region, higher frequency of mIPSC was observed in WT and LoF + EFV groups, compared to LoF group (*P* = 0.004 and *P* = 0.003, respectively). However, no significant difference was observed between WT group and WT + EFV group (*P* = 0.240) (Figure 6B).

Furthermore, the cumulative probability of peak amplitude and interevent interval were also compared (Figures 6C, D). The cumulative probabilities of inter-event interval among the groups were significantly different, while no significant difference was observed in the cumulative probability of peak amplitude.

## 4. Discussion

N-methyl-D-aspartate receptors have been shown to play a key role in normal brain functions, such as learning, memory, and brain development. They have also been implicated in the pathogenesis of many diseases, such as stroke, epilepsy, Alzheimer’s disease, Huntington’s disease, and schizophrenia (Dobrek and Thor, 2011; Lakhan et al., 2013; Zhou and Sheng, 2013). Although NMDAR mutations have been documented in patients with various neurological disorders, epilepsy is one of the most common diseases associated with NMDAR mutations. NMDAR acts as a receptor for excitatory amino acids, and its subunit gene mutations are closely related to epilepsy. Clinical case reports and cellular studies have shown that both NR gain-of-function and loss-of-function mutations can cause seizures (XiangWei et al., 2018). However, there is a paucity of animal studies to verify the function of the mutant sites of NMDAR, such as V685G. In addition, epileptic characteristics of GluN2A/GRIN2A-V685G mutant mice have not been reported. The present study investigated the influence of GluN2A/GRIN2A-V685G mutation on the epileptic features in mice. In this study, none of the mutant mice exhibited spontaneous seizures. We constructed a mouse model of hyperthermic seizure and found that the latency to seizure was significantly shorter in mice with GluN2A/GRIN2A-V685G mutation than in WT mice, indicating that the mutation made the animal more prone to seizures. EFV was found to relieve seizure occurrence and may be a potential therapeutic candidate for epilepsy, which was induced by NMDAR mutation.

Although GluN2A/GRIN2A-V685G mutation in mice induced much quicker seizures, as indicated by the shorter latency to seizure in mice of LoF group than mice of WT group, there were no significant differences between these two groups with respect to the intensity and duration of seizures. This implies that the seizure in mutant mice was not more serious than that in control mice. GluN2A/GRIN2A-V685G mutation may mainly cause susceptibility to epileptic seizures, and the intensity and duration of epileptic seizures may also be related to other factors in whole brain neural network. Further studies are required for in-depth characterization of the underlying mechanism.

Pyramidal neurons and interneurons are the main neurons in the hippocampal CA1 region. Pyramidal neurons are primary neurons, which can not only release excitatory neurotransmitters (such as glutamate) to transmit excitatory signals, but also receive excitatory or inhibitory synaptic signals from other neurons. Many kinds of interneurons have an inhibitory effect on pyramidal neurons. GABAergic interneurons account for approximately 10% of the total number of neurons (Freund and Buzsáki, 1996). The axonal synapses of these interneurons are distributed in various parts of pyramidal neurons and immediately release IPSC to regulate the discharge activity of pyramidal neurons. Interneurons also receive excitatory synaptic input from pyramidal neurons. These mechanisms modulate the excitatory/inhibitory balance of the neural network. Enhancement of excitability or the weakening of inhibition will cause imbalance of electrical activity resulting in abnormal discharge (Negrete-Díaz et al., 2007, 2018; Lyon et al., 2011; Falcón-Moya et al., 2021). As the receptor of excitatory amino acids, most of NMDAR is located on the postsynaptic membrane of pyramidal neurons and plays a dominant role in the generation of EPSC. LoF mutation in NMDAR would lead to EPSC reduction, inducing imbalance of neural network. In our study, GluN2A/GRIN2A-V685G mutation caused significant reduction in NMDAR-mediated current at the cellular level; cortical EEG revealed significant abnormal cortical discharge in mice with GluN2A/GRIN2A-V685G mutation. We hypothesized that this may be related to the distribution and function of extra-synaptic NMDAR. GluN2A/GRIN2A was shown to be expressed in both glutamate and GABAergic neurons (Bagasrawala et al., 2017). Therefore, changes in GluN2A function caused by *Grin2a* mutation may have different effects in different neurons.

Loss-of-function mutations have been speculated to attenuate IPSC by weakening the release of GABA from the presynaptic membrane of GABAergic neurons other than postsynaptic mechanism, leading to epilepsy (Gu et al., 2016; Gao et al., 2017). In our study, patch clamp was used to detect the current amplitude and maximum current area of NMDAR in the hippocampal CA1 region of mice. The current amplitude and maximum current area mediated by NMDAR in mice with GluN2A/GRIN2A-V685G mutation were significantly lower than those in WT mice. These results suggest that loss of NMDAR function in synapses affects the level of EPSC. However, compared with WT mice, mice with GluN2A/GRIN2A-V685G mutation showed no obvious change in action potential input resistance of pyramidal neurons, but showed decreased membrane potential and increased root number of the same current stimulus action potential, suggesting elevated excitability of mutated pyramidal neurons. This finding seemed to be contrary to the decreased EPSC. To clarify this, we further examined mIPSC in GABAergic interneurons, and found that the peak amplitude of mIPSC in mutation group was not significantly different from that in the WT group, but the distribution of frequency was decreased significantly. These results suggested that *GluN2A/Grin2a* mutations may affect the release of GABA in the presynaptic membrane of GABAergic interneurons, but not the GABA receptors in the postsynaptic membrane, although a reduction in mIPSC frequency does not necessarily mean a decrease in presynaptic GABA release. Studies have shown that GluN2A/GRIN2A-V685G mutation has a more obvious inhibitory effect on the function of extrasynaptic NMDAR (Swanger et al., 2016). In our patch clamp experiment, GluN2A/GRIN2A-V685G mutation was found to reduce the currents of EPSC and mIPSC in the hippocampal CA1 region. Combined with the results of cytological studies, we think that GluN2A/GRIN2A-V685G mutation had a stronger inhibitory effect on GABAergic neuron-mediated IPSC than on NMDAR-mediated EPSC within synapses, resulting in the imbalance of excitatory/inhibitory balance of the neural network in the hippocampal CA1 region, finally leading to abnormal discharge and seizures. Although, a recent study has shown that loss of NMDARs can also cause a decrease in IPSC by postsynaptic mechanisms, regulating GABAergic synapse development, but mainly *via* the CaM-binding motif in the C0 domain of the NMDAR GluN1 subunit rather than GluN2A (Gu et al., 2016). Further studies are required for in-depth characterization of the underlying mechanism, such as the expression and function of GABA receptors.

Some mutations have been shown to induce decreased expression levels of NMDAR, resulting in decreased function (Swanger et al., 2016). However, in this study, mutation did not alter the mRNA or protein expressions of GluN2A. In fact, the level of GluN2A mRNA showed a slight increase in the LoF group compared with the WT group. In previous studies, most of the mutations causing decreased expression of NMDAR occurred in conservative Cys residues, and the disulfide bonds formed could form a stable ring structure on the interface of heterodimer and promote the aggregation of GluN1 and GluN2 subunits, reducing the levels of NMDAR on the cell surface (Lee et al., 2014; Serraz et al., 2016). In addition, some mutated residues of NMDAR were in the charged region of the dimer interface, and the charge conversion of glutamate may hinder the interaction between subunits, inducing failure of the subunit to fold or assemble properly, resulting in reduced protein level and receptor transport (Karakas and Furukawa, 2014). GluN2A/GRIN2A-V685G was in the S2 region of the ABD domain, not in the above region, which did not affect the expression and localization of NMDAR, but weakened the effectiveness of glutamate due to the influence of binding ability with ligand. The increased expression of GluN2A mRNA in the mutant mice may be related to the compensatory expression after the decline of NMDAR function, which needs more studies.

Tang et al. investigated the function of drugs targeting this mutant site at the cellular level. They found that 24(S)-HC and pregnenolone sulfate (PS) had positive allosteric regulation on GluN2A/GRIN2A-V685G mutations (Tang et al., 2020). 24(S)-HC is a metabolite of cholesterol and plays an essential role in the brain. Excess cholesterol in the brain is transformed into 24(S)-HC by cholesterol 24S-hydroxylase (cytochrome P450 46A1, CYP46A1). It can cross the blood brain barrier and be metabolized in the liver (Dietschy and Turley, 2004). CYP46A1 is a key rate-limiting enzyme that regulates the level of cholesterol and 24(S)-HC in brain. Studies have shown that EFV can enhance the activity of CYP46A1 *in vitro* and *in vivo* (Mast et al., 2014, 2017; Anderson et al., 2016). Besides, EFV has been introduced into the research of Alzheimer’s disease in recent years (Mast et al., 2014; Anderson et al., 2016). Based on these, we used EFV to restore the function loss of NMDAR caused by GluN2A/GRIN2A-V685G mutation. The results showed that EFV intervention prolonged the latency of hyperthemic seizure in mutant mice, indicating an inhibitory effect of EFV on epileptic seizures induced by mutation. *In vivo* EEG examination showed inhibition of cortical EEG discharge after EFV intervention. Patch clamp studies at the brain tissue level showed that EFV intervention induced an increase in NMDA-mediated EPSC, and the spontaneous potential frequency of mIPSC. The action potential threshold of pyramidal neurons in the hippocampal CA1 region was also increased. Thus, EFV may restore the function of GluN2A/GRIN2A-V685G, a type of NMDAR with loss of function, promoting the neuronal network excitation/inhibition balance, thus inhibiting seizures. Till date, the application of EFV in the nervous system has only been reported in Alzheimer’s disease, but not in epilepsy. Further investigation should be conducted to extend the clinical application of EFV in epilepsy and other related diseases caused by the mutation of NMDAR. This can provide a theoretical basis and inform future research direction for precision treatment of inherited forms of epilepsy and other related genetic diseases. However, this subject also has the shortcomings of insufficient sample size and difficulty in quantification of cortical EEG *in vivo*, which need to be further improved.

## 5. Conclusion

We constructed a hyperthermic seizure model in GluN2A/GRIN2A-V685G mutant mice, and found that GluN2A/GRIN2A-V685G mutation can change the features of epileptic seizure, rendering animal more prone to seizures. Furthermore, EFV, an anti-HIV drug, was found to restore the function of NMDAR and alleviate epileptic seizure, indicating its potential use for the treatment of epilepsy.

## Data availability statement

The raw data supporting the conclusions of this article will be made available by the authors, without undue reservation.

## Ethics statement

All experimental procedures involving mice were approved by the Animal Welfare and Research Ethics Committee of The First Hospital of Jilin University (Permit Number: 20200721).

## Author contributions

TZ: funding acquisition, investigation, methodology, and writing—original draft. WL: funding acquisition, project administration, supervision, and writing—review and editing. SF: supervision, formal analysis, and writing—review and editing. YD: investigation, software, and visualization. RZ: data curation, visualization, and methodology. XZ and GL: investigation and methodology. CZ: resources and supervision. All authors contributed to the article and approved the submitted version.
