# Supplementary figures and images for: Efavirenz restored NMDA receptor dysfunction and inhibited epileptic seizures in GluN2A/Grin2a mutant mice
Source: Front Neurosci. 2023 Mar 2;17:1086462. doi: 10.3389/fnins.2023.1086462 (PMC10017539; doi:10.3389/fnins.2023.1086462)

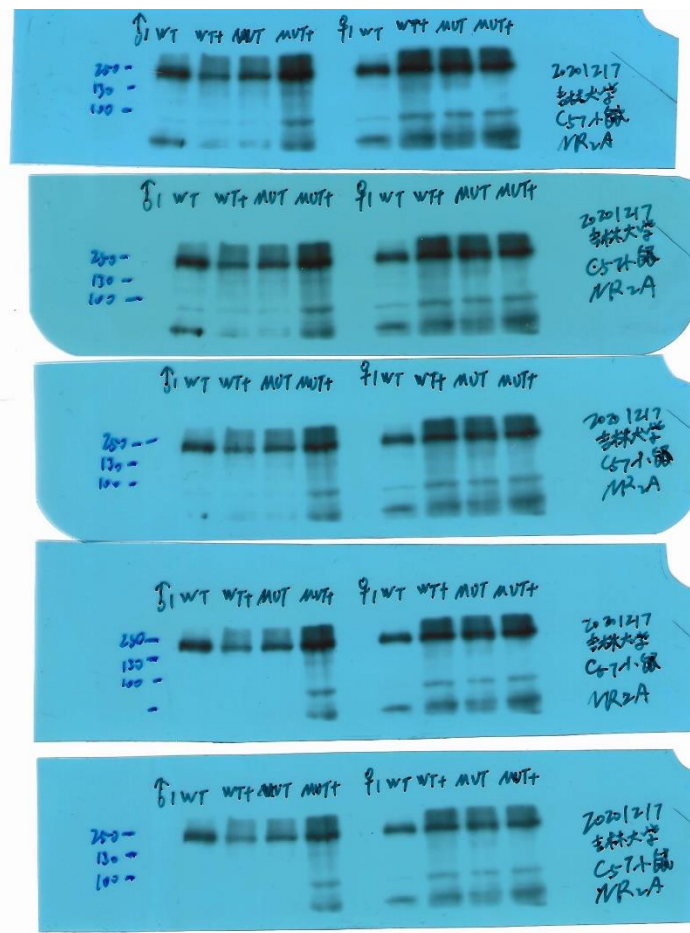

20201217, NR2A ♂ 1 ♀ 1-165KDa

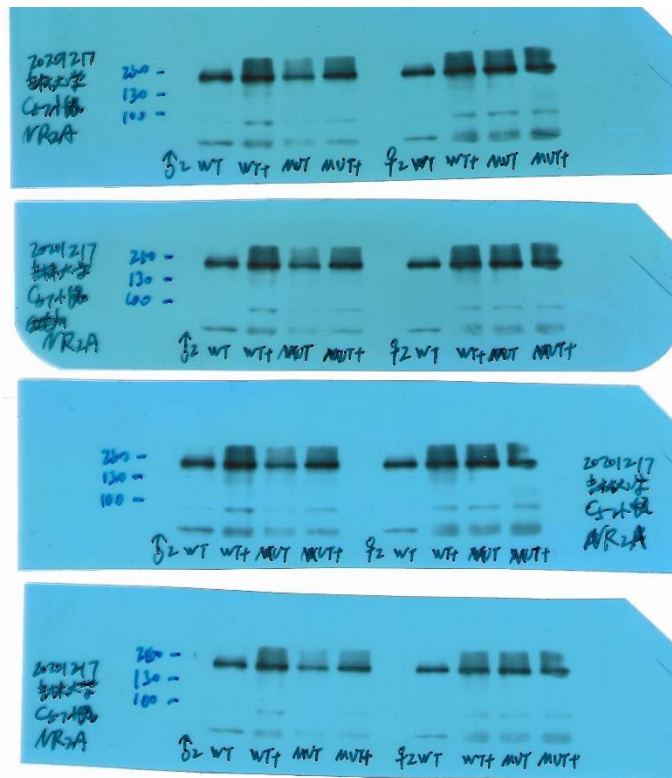

20201217, NR2A ♂ 2 ♀ 2-165KDa

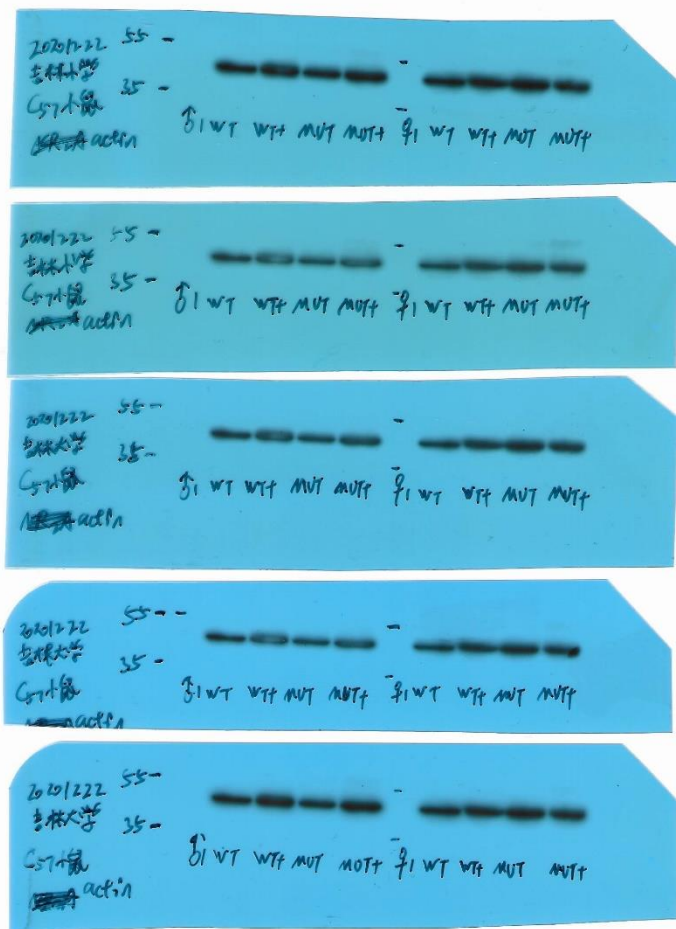

20201222, Actin ♂ 1 ♀ 1

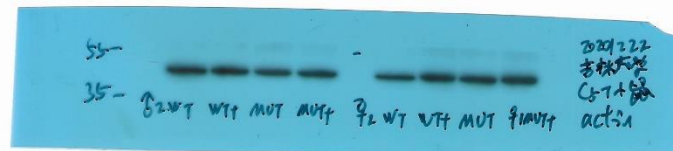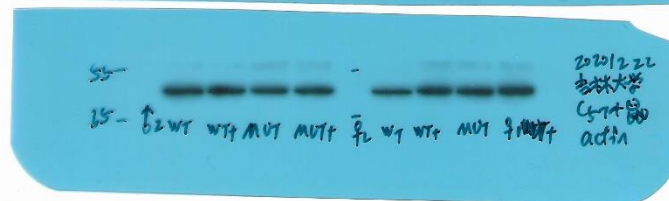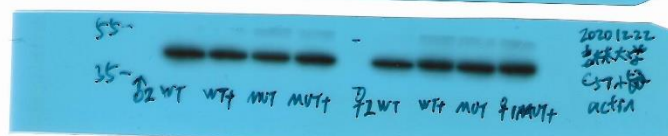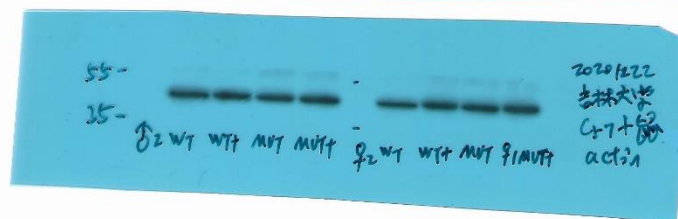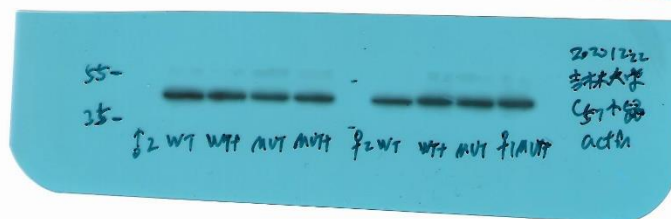

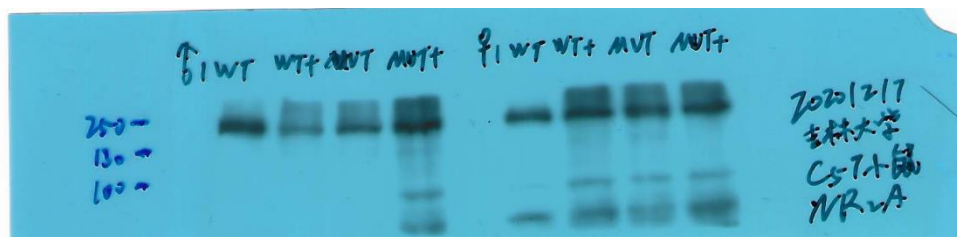

NR2A,1 1

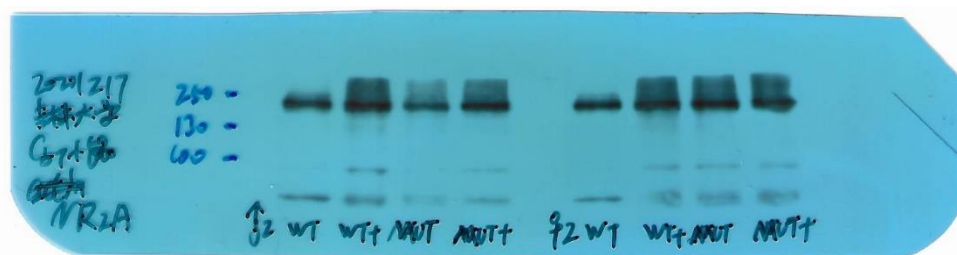

NR2A,2 2

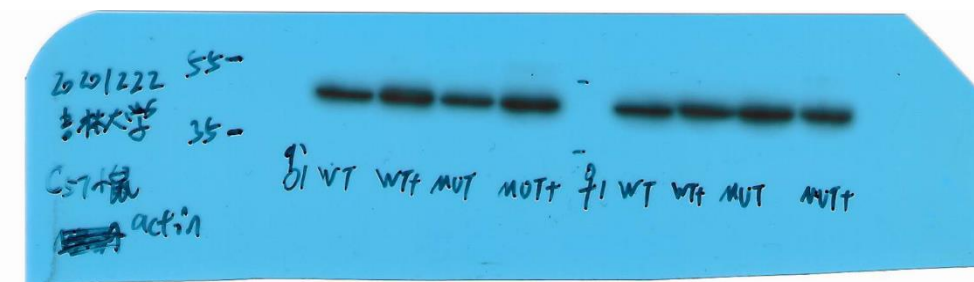

$\beta$ -Actin,1 1

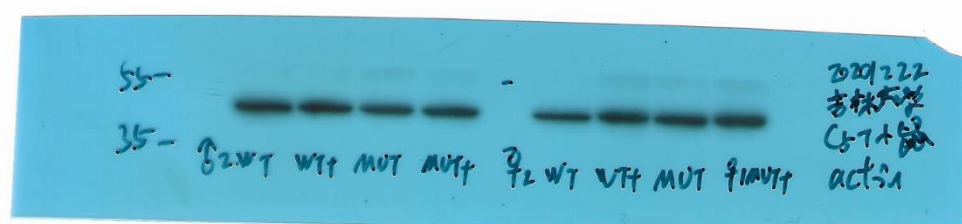

$\beta$ -Actin,2 2

Supplement: Supplementary file 1 [file Data_Sheet_1.PDF]
